# Spaces that move children: The role of the physical environment in developing motor competence and physical literacy

**DOI:** 10.1371/journal.pone.0350957

**Published:** 2026-08-20

**Authors:** Javier Molina-García, Xavier García-Massó, Ana Queralt, Nuria Ortega-Benavent, Cristina Menescardi, Isaac Estevan

**Affiliations:** 1 AFIPS Research Group, Department of Teaching of Physical Education, Arts and Music, University of Valencia, Valencia, Spain; 2 Epidemiology and Environmental Health Joint Research Unit, FISABIO-UJI-UV, Valencia, Spain; 3 AFIPS Research Group, Department of Nursing, University of Valencia, Valencia, Spain; Polytechnic University of Guarda, PORTUGAL

## Abstract

The significant relationship between attributes of the physical environment of schools and residential neighborhoods with children’s physical activity (PA) has been well documented in the literature. However, there is a lack of research investigating the relationship between the characteristics of physical environment and motor competence (MC) as well as physical literacy (PL). This study explores how the physical environmental factors in schools and residential neighborhoods moderate the relationship between PA, MC and perceived PL (PPL) in children. A model-based tree approach was applied to a sample of 396 school-aged children (51.5% girls; mean age = 10.3 ± 5.8 years) to examine specific environmental features and their potential to moderate the associations between PA and MC, as well as PA and PPL. Environmental characteristics were assessed using both an audit tool and a questionnaire. PA and PPL were measured via self-reported questionnaires, while MC was evaluated through a standardized physical performance test. The findings revealed that the school sports/play facilities, aesthetics and PA policies, alongside neighborhood recreational facilities, significantly amplify the strength of the above-mentioned relationships. Specifically, the positive association between PA and MC was strongest in schools with better facilities and high aesthetic scores (slope *b* = 2.07 vs. *b* = 0.86 in lower-quality settings). Similarly, the relationship between PA and PPL was most pronounced when supportive school policies and greater availability of neighborhood recreational infrastructure were both present (*b* = 0.40 vs. *b* = 0.24). The model-based trees outperformed traditional regression models by providing higher predictive accuracy (improving Pearson correlation by r = 0.06–0.09) and offered a nuanced view of how place-based factors interact to affect children’s development. The findings emphasize the importance of designing equitable school and neighborhood environments to support children’s motor skills, and selected dimensions of PL, as well as their engagement in PA.

## Introduction

Fostering lifelong physical activity (PA) and overall well-being in children is a critical priority in both public health and education, which requires a deep understanding of foundational constructs such as motor competence (MC) and physical literacy (PL) [1–7]. MC refers to proficient motor-skill performance, including coordination and motor control [1]. MC is a multidimensional construct encompassing various motor skills and their underlying mechanisms. It plays a pivotal role in influencing various aspects of children’s health, including their physical fitness, psychological well-being and overall health-related quality of life [2–4].

A holistic concept related to individual health is PL, which is understood as an integrated construct spanning physical, psychological, social and cognitive domains [5], rather than a set of isolated components. In this view, fostering PL entails attending simultaneously to physical competence, motivation and confidence, knowledge and understanding, and the value and commitment to engage in lifelong PA [6,7]. Perceived PL (PPL) specifically refers to how children view their own PL [8], such as evaluating their own physical competence, motivation and confidence, and their knowledge and understanding in participating in PA [9]. Indeed, PL is a broader construct than MC covering not only proficiency in motor skills but, as mentioned encompasses various aspects of an individual’s ability to engage in PA [10]. Both PPL and MC can impact children’s participation in PA [11] and improve their overall health outcomes, essential for maintaining a healthy lifestyle [12].

The literature has established a strong link between PA participation and both MC [13,14] and PL [15]. This relationship is often described as dynamic and reciprocal, suggesting that higher levels of PA are related to better children’s MC and PL development [15–18] and is crucial, as MC and PL are determinants of lifelong PA and overall health [15,19].

MC is associated with numerous types of factors such as biological and demographic, behavioral, cognitive, emotional, and psychological, cultural and social factors, creating a system with adaptable components that work together synergistically and cooperatively to generate a diverse array of motor results [20]. The relationship between MC and PA is also complex and influenced by a variety of correlates such as gender, physical fitness, perceived MC and physical environmental factors [13,21,22]. It is now generally accepted that the physical environment plays a crucial role in facilitating both children’s and adolescents’ PA behavior [23–26]. For instance, positive attributes of the home neighborhood, such as the presence of recreational facilities, higher socio-economic status (SES) and a safe transport infrastructure encourage PA, whereas negative factors such as traffic and safety concerns hinder it [23–26].

Recent studies have highlighted the importance of the physical environmental factors in promoting not only PA but also MC [27–29]. Although the school environment is considered an important factor in PA behavior, as children spend a significant amount of time at school [30], little is known about how environmental characteristics at home and school influence this relationship. In particular, the moderating effects of perceived dimensions, such as PPL, remain underexplored. In fact, the relationship between PA and MC can be moderated by a variety of physical environmental factors, including geographical location and the school environment [27–29]. It seems that children from rural areas have better MC and spend more time outdoors playing than those children from urban areas [28], while less densely populated areas may provide more opportunities for PA and MC development. In relation to the school physical environment, the presence of sports and play facilities and well-designed school grounds (including aesthetics) support MC [27,29]. Further research is still needed to deepen the analysis of the relationships between the neighborhood and school characteristics and the development of MC by considering children’s PA behavior. Future research should also examine the role of environmental factors in PL due to the lack of information on this topic [31].

The relationship between PA, MC, and PPL is a critical area of research in education and public health, while understanding how physical environmental factors moderate these relationships can provide valuable insights for developing effective interventions and policies. The aim of this study was thus to examine the relationship between PA, MC and PPL in a school-age population, considering through a model-based tree approach the physical environment in the school and home-neighborhood as factors that moderate these relationships. We hypothesize that PA is positively associated with MC and PPL, but that these relationships are heterogeneous across the sample. Specifically, we expect that environmental factors in the school and home-neighborhood identify subgroups where the impact of PA on MC and PPL is significantly moderated. By examining the study objective, we considered it would be possible not only to determine whether there are subsamples in which the influence of PA on the outcome variables differs, but also to identify the environmental variables that actually affect this relationship. With this information, recommendations can be made to the relevant stakeholders to maximize the impact of PA on MC and PPL while considering the physical environment.

## Methods

### Participants

#### Recruitment and timeline.

The sample was recruited in two waves as part of the ALPHYL (2022–2023) and ALPHYL 2.0 (2023–2024) studies [32]. The recruitment period for the first wave was from 05/12/2022 to 20/01/2023, whereas the recruitment period for the second wave was from 04/12/2023 to 19/01/2024. The sample was selected from nine primary schools of the city of Valencia (Spain) and its metropolitan area.

### Sample characteristics

#### Children sample.

Initially, the sample consisted of 864 children, whose parents or guardians provided signed informed consent before the study. The final analyzed sample included 396 children (51.5% girls; mean age = 10.33 years, SD 0.80). This reduction from the initial group was due to the exclusion of children whose parents did not complete the parental questionnaire, or this questionnaire contained incomplete information or omissions. To ensure a fully inclusive environment, all students were invited to participate in the proposed activities regardless of physical or mental difficulties, thereby avoiding social exclusion within the school setting. However, data from students with evident physical or mental disabilities that precluded the standardized completion of the assessments were excluded from the statistical analyses. This exclusion was necessary to maintain the internal validity of the study, as the assessment tools employed were not specifically validated for these populations. We acknowledge that while this ensures statistical homogeneity, it also limits the generalizability of our findings to children with diverse functional needs.

#### Parents sample.

A total of 404 parents or guardians completed a questionnaire consisting of socio-demographic variables and the environmental characteristics of their neighborhoods. After excluding eight participants due to incomplete information or omissions, the final parental sample consisted of 396 individuals (75% women; mean age = 42.88 years, SD 5.81).

### Ethics statement

The procedures were performed in accordance with the Helsinki Declaration, and the study was approved by the Ethics Committee of Research in Humans of the University of Valencia (reference 1259844).

### Procedures

As described in the ALPHYL study protocol [32], the researchers first contacted the school principals to explain the study. Once they approved their participation, the families were informed of the project’s characteristics. The measurement protocol included three sessions: in the first, the children completed a questionnaire on their PA participation and PPL. In the second, the students performed the Canadian Agility Movement Skill Assessment (CAMSA) test [33]. In another session, the researchers conducted an audit of the schools’ physical characteristics in relation to PA. The parents also completed a questionnaire on socio-demographic factors (their own and their children’s age and gender, highest level of education in the family as a proxy of SES, etc.) and on the characteristics of the home-neighborhood.

### Instruments

#### Physical environment: school environment and home-neighborhood.

The International Study of Childhood Obesity, Lifestyle and Environment [34] and School Audit Tool (ISAT) [35] were used to evaluate the school PA environment. The ISAT tool can be used to conduct reliable objective audits of the school environment across international school settings [35]. The ISAT tool is divided into two sections: the schools’ built environment and its food environment, although in the present study we only used the section on the built environment.

The subscales directly related to assessing the school’s physical characteristics were used, i.e.,: sports and play facilities (9 items), aesthetics (6 items), suitability of school grounds (3 items), and other facility provision (5 items). The sports and play facilities subscale evaluates the availability and quality of facilities such as playgrounds and sports fields. This subscale originally had 8 items; however, the research team decided to include an additional item regarding the presence of indoor sports facilities (e.g., gymnasium) to better capture the Spanish school context. Based on our contextual knowledge, indoor facilities are a common feature of local schools and represent a key space for PA. The inclusion of this item was therefore necessary to ensure the ecological validity of the instrument within the local cultural setting. Aesthetics items were related to the presence of aspects such as flowerbeds, ambient noise and murals/outdoor art, which could contribute to the aesthetic appeal of school grounds for PA. The school grounds were evaluated for their suitability in relation to sport, informal games and general play, while the items dealing with other facility provisions assessed, for instance, the availability of drinking fountains and wildlife/nature gardens. The ISAT questionnaire includes different types of responses to the items, as well as different processes for calculating the scores of each variable [35]. Regarding the scoring protocol, items within the ISAT were scored based on presence (e.g., 0 = No, 1 = Yes), availability, or quality (using Likert-type scales ranging from 1 to 4). For analysis, answers were dichotomized to correspond to ‘present and functional’ (1) versus ‘present and not functional or not available’ (0). Following the original instrument’s guidelines [35], each subscale score was calculated as the sum of the items. Higher scores in each subscale indicate a more favorable school physical environment for PA. In the present study two evaluators from the research team separately evaluated two schools to obtain a degree of agreement ≥ 95%. After the independent evaluation of the two schools, 97.6% of agreement was obtained. The remaining schools were then evaluated by one of the two evaluators. All the evaluations were conducted in person at the schools.

As in many cases the schools included the modification of the physical environment, their PA policies were also evaluated, e.g., in the case of programs for the organization of recess time, whether they involved the use of mobile equipment for children to use in the playground [25]. Considering previous work by Woods et al. [36], the research team assessed the number of policies in 9 areas: whole school PA, sport/extracurricular, active breaks/recess, PA in the classroom, physical environment, shared-use agreements, active transport, surveillance and physical education. For this, a researcher interviewed one of the school principals and collected information on the use of PA policies in each of the areas analyzed. Finally, a single variable with the total number of school policies was calculated for each school.

The parents completed the International Physical Activity and the Environment Network (IPEN) version of the Neighborhood Environment Walkability Scale for Youth (NEWS-Y-IPEN) [37] to measure home neighborhood attributes. This instrument has good factorial and construct validity and was developed to facilitate international comparability of different studies [37]. The NEWS-Y-IPEN is based on eight subscales: residential density, land use mix, recreational facilities, accessibility and walking facilities, traffic safety, pedestrian infrastructure and safety, precautions against crime and aesthetics.

In relation to residential density, the parents indicated the frequency of six types of homes in their neighborhood. The response options ranged from 0 (none) to 4 (all). The six items referred to: detached single-family residences (1); multiple-family buildings with 1–3 stories (11); multiple-family buildings with 4–6 stories (25); multiple-family buildings with 7–12 stories (50); multiple-family buildings with 13–20 stories (75); and multiple-family buildings with over 20 stories (100). These items were weighted and the scores summed (weightings are shown in parentheses). Higher scores meant higher residential density.

As a measure of land use mix, the parents reported how long it would take them to walk to 13 different types of destinations from their home: convenience store or equivalent; supermarket; laundry/dry cleaner; library; post office; bank/credit union; pharmacy/drug store; any school; the child’s school; fast food restaurant; coffee shop; non-fast food restaurant; and transit stops (bus, subway, and train). Response options were: 1–5 minutes (1); 6–10 minutes (2); 11–20 minutes (3); 21–30 minutes (4); 31 + minutes (5); don’t know (5). The items were reverse-scored and the land-use mix diversity score was computed by averaging the score for the 13 destinations. A recreational facilities score was computed by averaging six of these types: indoor recreation/exercise facility; beach/lake/river/creek; bike/hiking/walking trails/paths; basketball court; other playing fields/courts (e.g., soccer, skate park, etc.); and swimming pool. A park-proximity score was computed by averaging responses for two items, i.e., small public park and large public park. Higher scores for land use mix, recreational facilities, and proximity to parks indicated better access to these different types of destinations.

The following subscales were also created by averaging different items: accessibility and walking facilities (5 items: hilly streets, fewer cul-de-sacs, many different routes, sidewalks available, and separated sidewalks); pedestrian infrastructure and safety (3 items: lighting, visibility of walkers/bikers from homes, and crosswalks/signals); traffic safety (3 items: difficult/unpleasant to walk because of traffic, traffic speed, and drivers exceed speed limit); safety from crime (4 items about the fear of a child being hurt by a stranger in different situations); and aesthetics (3 items: interesting things, beautiful/natural things, and visually attractive buildings/homes). The type of response to these subscales was based on a Likert scale from 1 (strongly disagree) to 4 (strongly agree). Answers to corresponding items were averaged to compute each subscale (reverse scored as needed). Higher scores indicated higher walkability, safety, and aesthetics.

### Physical activity

The Spanish version [38] of the Physical Activity Questionnaire for older children (PAQ-C) was used to assess PA behavior [39]; this is a self-reported 7-day recall questionnaire that assesses children’s sports practice (e.g., “Have you done any of the following activities …? If yes, how many times?”) and the level and frequency of PA during different periods of the day: school recess (e.g., “… what did you do most of the time at recess?”), physical education, lunch break, after school, in the evenings, and at weekends. The questionnaire is composed of nine items and the responses are scored on a five-point Likert scale ranging from 1 (low) to 5 (high). The average score of all items gives an indicator of from 1 to 5 on the overall PA level. An extra item that was not used to calculate the average score is used to assess whether the participant was not able to engage in the usual PA due to, for instance, being ill in the previous week. For those children reporting illness, the PAQ-C was re-administered two weeks later. In a recent study among Spanish children [40], the PAQ-C showed good construct validity and reliability. In the present study, Cronbach’s alpha was 0.76 for the overall questionnaire

### Motor competence

The Canadian Agility and Movement Skill Assessment (CAMSA) was used to evaluate the children’s MC in the form of the fundamental and combined movement skills [33]. This test has three components: three two-footed jumping, sliding a distance of 3-m, returning, catching a ball, throwing a ball accurately (at a wall-mounted target 5 m away), skipping 5 m in a rhythmic pattern, performing a hopping in and out of six hoops, and kicking a ball between two cones 5 m apart. The final result of the CAMSA involves two key criteria: the quality of the movement pattern executed or the motor skill score (skill performance); and the total time taken to complete the circuit (completion time). This latter is transformed into a point score (i.e., time score), ranging from 1 to 14 [41]. The quality of the movement is evaluated by 14 skill performance criteria, ranging from 1 to 14 with one point awarded for each criterion completed correctly [41]. Combining the skill performance and completion time scores, a total CAMSA score of 28 points is obtained [41]. Higher values indicate better agility and coordination. Each participant completed the CAMSA on two separate occasions: the first familiarization time twice; the second time of formal assessment also twice. Similar to previous studies [33,40], two raters were involved in coding the recorded videos; inter-rater reliability was excellent for time scores (ICC = 0.99), item scores (0.84, 95% CI [0.75, 0.90]), and total CAMSA scores (ICC = 0.95, 95% CI [0.86, 0.96]). The best score of the two formal assessment trials was considered for the present study.

### Perceived physical literacy

PPL was evaluated by a self-report using the PL-C Quest [8]. Each item consisted of two pictograms and four answer options, with lower scores indicating lower levels of PPL in each domain. The questionnaire has 30 items grouped into four domains: physical (12 items); psychological (7 items); social (4 items); and cognitive (7 items). For example, in the physical domain, the child is asked how good he/she is at 12 tasks such as jumping on one leg or running very fast. Responses were based on a four-point Likert scale from 1 (Not very good) to 4 (Very good). The average score of all items provided an indicator of the overall individual PPL. There is evidence of the validity (i.e., concurrent, predictive, and structural validity) of this questionnaire in Spanish children and adolescents with an adequate reliability and good-to-excellent test-retest reliability [42]. In the present study, Cronbach’s alpha was 0.91 for the overall questionnaire.

### Data analysis

All the analyses were conducted in R (version 4.4.1) within the RStudio environment (version 2024.04.2 + 764). After loading the dataset, we addressed the missing data using a k-nearest neighbors approach (k = 3), based on a distance metric (Euclidean distance) in the space of non-missing variables and used their observed values to estimate the missing entry [43]. The chosen k value was intended to preserve local structure while preventing over-smoothing, which can occur with larger numbers of neighbors.

Following imputation, we focused on understanding how environmental factors (from home-neighborhood and school context) might moderate the relationship between our primary predictor (i.e., PA) and the outcome variable (i.e., see MC, PPL, and PL domains in the supporting information), controlling the models by gender and SES. To capture potential subgroup differences, we used a model-based tree framework implemented through the *mob()* function in the *partykit* package. This procedure recursively partitions the data based on selected environmental variables (e.g., Residential density and Land use mix), fitting a node-specific linear model for the output variable using PA as the predictor and controlled by gender and SES in each partition [44]. Notably, all school- and home-neighborhood-related variables were entered into the models simultaneously. This allowed the algorithm to treat these environments as co-existing factors, identifying potential interactions across different domains rather than analyzing them in isolation. In other words, while the outcome and principal predictor remain consistent across the entire dataset, the tree algorithm divides the observations into subgroups where local regressions better explain variation in the output variable. We specified relatively conservative minimum node sizes (minsplit = 40) to ensure reliable estimation in each final node and set a significance level of α = 0.2 for splitting so as not to prematurely discard potentially meaningful interactions or moderating effects [44].

For comparison, we also fitted a conventional multiple linear regression using the same predictor without allowing any structural splits. To evaluate whether the model-based tree afforded clearer insights or better predictive accuracy, we calculated the mean squared error (MSE) and mean absolute error (MAE) for both approaches along with Pearson correlations between the observed and predicted values [45]. We then generated a visualization of the tree to display how the data were partitioned and examined individual terminal nodes to show how the relationship between PA and the output variables varied across environmental contexts. Scatter plots for each node overlaid with locally fitted regression lines offered an intuitive look at these subgroup-specific associations.

The chosen approach (i.e., model-based trees) was particularly suited to our investigation as it allowed us to: A. Detect heterogeneity: By splitting the sample according to environmental variables, we could test whether the effect of PA on the output variables differed across different contextual conditions, an important consideration in public health and environmental research. B. Preserve interpretability: Within each node we fitted a standard linear regression model, maintaining the interpretability of coefficients (e.g., effect sizes and direction) provided by a simple global linear model. C. Balance complexity and parsimony: We mitigated the risk of overfitting by specifying thresholds for splitting (minsplit) and a moderately liberal alpha level. This ensured that we captured potentially meaningful subgroups without producing a tree with too many, potentially spurious splits.

Overall, this methodology combines the strengths of parametric linear modeling (coefficient interpretability) with the flexibility of tree-based partitioning (context-specific relationships), making it a robust framework for exploring and interpreting complex interactions in our dataset. Furthermore, while PL is inherently a person-centered and holistic construct, we opted for this quantitative framework to identify shared environmental patterns that influence individual experiences. By employing model-based trees, we move beyond global averages to acknowledge the heterogeneity of PL, effectively capturing how different contexts (school and neighborhood) moderate the relationship between PA and PPL, as well as between PA and MC. This approach allows us to maintain a balance between the individualized nature of the PL construct and the need for scalable, evidence-based insights in both public health and educational research.

## Results

### Descriptive results

Table 1 gives the descriptive statistics of all the variables included in the present study, plus the differences between boys and girls in the variables. Results indicate significant gender differences in MC, PA, PPL, the physical domain of PL, recreational facilities, and park proximity. In all these variables, boys reported higher scores than girls. The magnitude of these differences was small for the majority of variables (0 < |d| < 0.5), except for MC, which yielded a medium effect size (d = 0.57).

**Table 1:** Descriptive data and gender comparisons.

	Total (*n* = 396)	Boys (*n* = 192)	Girls (*n* = 204)	*t*-student	*p*-value	*Cohen’s d*
Motor competence	18.9 (3.65)	19.93 (3.38)	17.91 (3.64)	5.7	<0.001	0.57
Perceived physical literacy	3.09 (0.50)	3.15 (0.52)	3.04 (0.48)	2.18	0.03	0.22
Physical domain	2.91 (0.57)	3.03 (0.56)	2.79 (0.56)	4.19	<0.001	0.43
Cognitive domain	3.29 (0.60)	3.30 (0.62)	3.28 (0.57)	0.4	0.69	0.03
Social domain	3.28 (0.68)	3.27 (0.69)	3.30 (0.68)	−0.42	0.67	−0.04
Psychological domain	3.09 (0.71)	3.12 (0.73)	3.06 (0.7)	0.82	0.41	0.08
Socio-economic status	4.99 (1.24)	4.99 (1.19)	5.00 (1.28)	−0.04	0.96	−0.01
Physical activity	3.15 (0.72)	3.31 (0.73)	3.00 (0.68)	4.4	<0.001	0.44
Residential density^a^	439 (157)	439.07 (162.35)	439.15 (151.43)	−0.01	0.99	−0.00
Land use mix^a^	1.99 (0.73)	2.01 (0.68)	1.98 (0.78)	0.5	0.62	0.04
Recreational facilities^a^	2.42 (0.69)	2.48 (0.67)	2.37 (0.71)	1.65	0.01	0.16
Park proximity^a^	1.76 (0.82)	1.84 (0.86)	1.67 (0.78)	2.03	0.04	0.21
Accessibility and walking facilities^a^	3.45 (0.49)	3.43 (0.50)	3.47 (0.48)	−0.7	0.48	−0.08
Pedestrian infrastructure and safety^a^	3.09 (0.67)	3.08 (0.69)	3.11 (0.66)	−0.33	0.74	−0.04
Aesthetics^a^	2.64 (0.81)	2.61 (0.84)	2.67 (0.79)	−0.65	0.52	−0.07
Safety from crime^a^	2.79 (1.01)	2.82 (1.02)	2.76 (1.01)	0.63	0.53	0.06
Traffic safety^a^	2.75 (0.71)	2.73 (0.71)	2.77 (0.72)	−0.59	0.56	−0.06
Sports and play facilities^b^	5.14 (1.12)	5.20 (1.11)	5.08 (1.12)	1.02	0.31	0.11
School aesthetics^b^	5.14 (0.55)	5.15 (0.53)	5.12 (0.57)	0.51	0.61	0.05
Suitability of school grounds^b,c^	3.00 (0.00)	3.00 (0.00)	3.00 (0.00)	--	--	0.00
Other facility provision^b^	3.33 (1.27)	3.36 (1.28)	3.29 (1.27)	0.55	0.58	0.05
Physical activity policies^b^	5.38 (1.72)	5.48 (1.75)	5.28 (1.69)	1.15	0.25	0.12

^a^Home-neighborhood.

^b^School environment.

^c^Zero variance was observed for this parameter; all cases recorded identical values, indicating a consistent baseline across the study sites.

### Model-based trees

**Fig 1:**
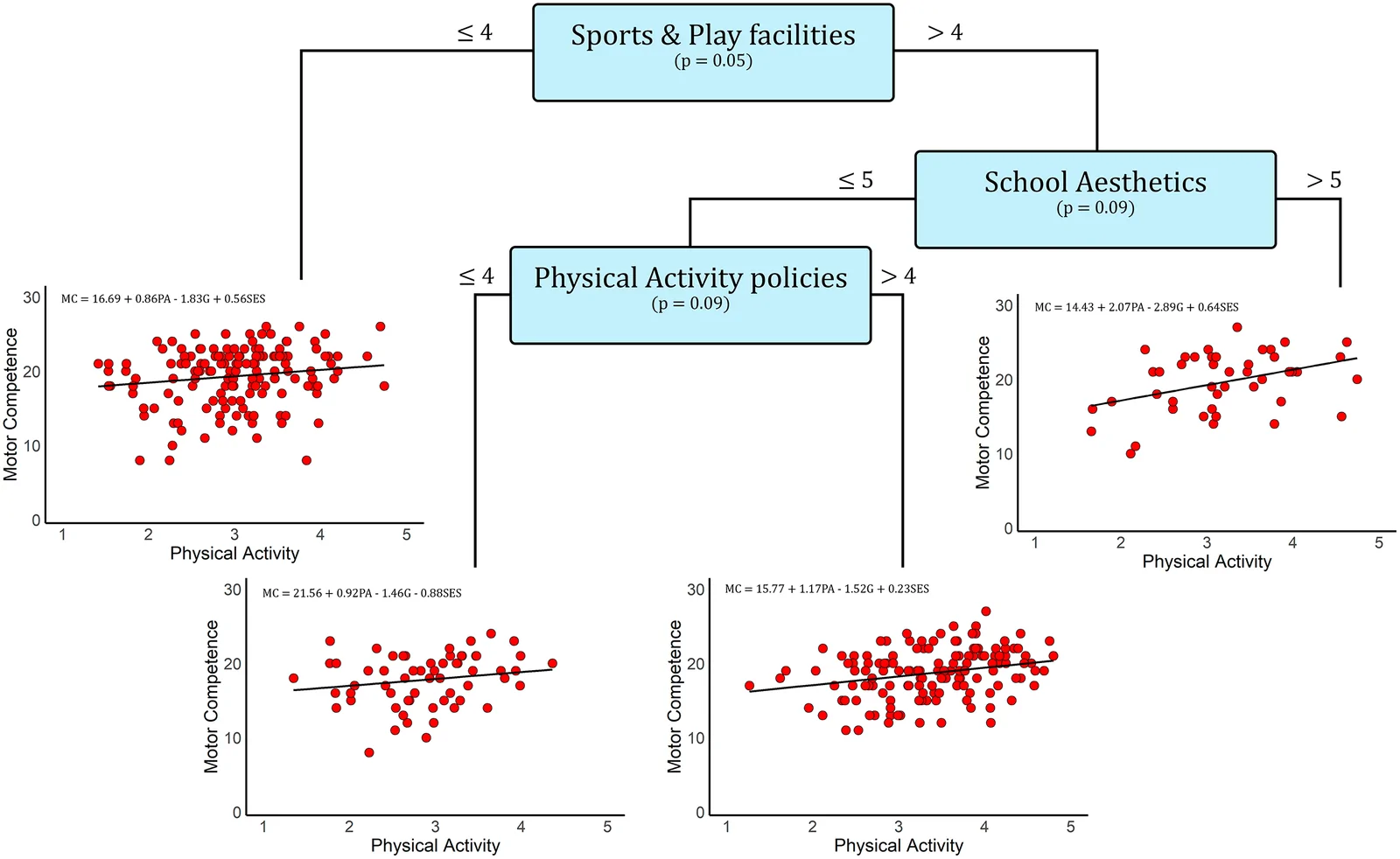
Linear regression-based tree for Motor Competence (MC). The terminal nodes of the tree plot report the intercept and estimate of physical activity (PA), but the models are controlled by gender (G) and socio-economic status (SES) as reported in equations. Variables related to the school environment are shown with a blue background, while those related to the home neighborhood are shown with a yellow background.

To address our primary objective of identifying how environmental factors moderate the impact of PA on MC and PPL, we employed model-based recursive partitioning. The model-based tree for MC is presented in Fig 1. Three school variables emerged as potential mediators (i.e., Sports and play facilities, School aesthetics, and PA policies) leading to four distinct subsamples with different slopes for the PA/MC relationship. Among the participants in schools scoring ≤4 in Sports and play facilities (*n* = 147), the slope was *b* = 0.86, representing the baseline relationship where PA contributes to MC, but its impact is limited by the lack of adequate infrastructure. For those in schools where Sports and play facilities >4, School aesthetics ≤5, and PA policies ≤4 (*n* = 63), the slope increased to *b* = 0.92. Students attending schools with Sports and play facilities >4, School aesthetics ≤5, and PA policies >4 (*n* = 142) showed a slope of *b* = 1.17, suggesting that the implementation of formal PA policies further boosts the effectiveness of PA on motor development. Finally, the participants in schools with over 4 points in Sports and play facilities and >5 on School aesthetics (*n* = 44) exhibited the steepest slope (*b* = 2.07). In practical terms, this indicates that the impact of PA on motor development is more than doubled in optimal school environments. These results suggest that high-quality facilities and aesthetics act as a catalyst, significantly amplifying the benefits of PA for children’s MC.

**Fig 2:**
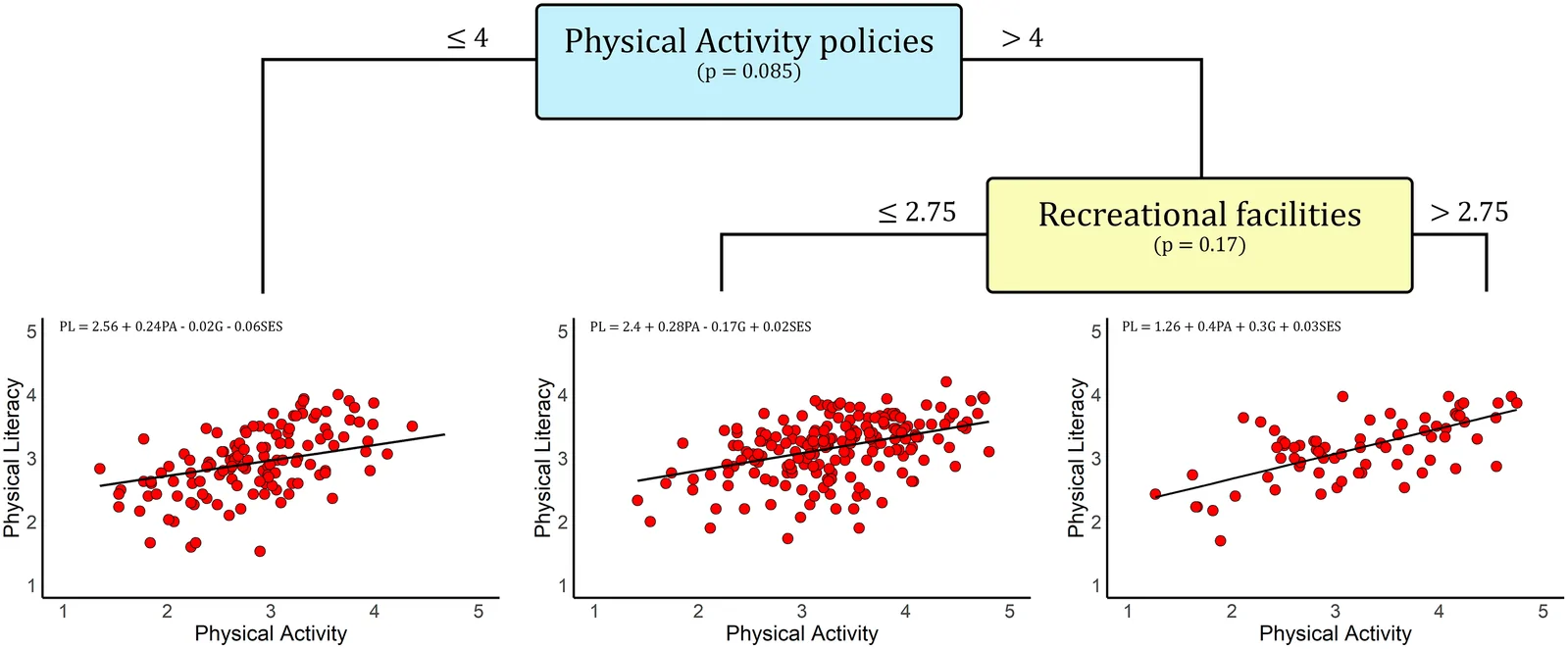
Linear regression-based tree for Perceived Physical Literacy (PPL). The terminal nodes of the tree plot report the intercept and estimate of physical activity (PA), but the models are controlled by gender (G) and socio-economic status (SES) as reported in equations. Variables related to the school environment are shown with a blue background, while those related to the home neighborhood are shown with a yellow background.

Three subgroups were identified in the recursive partitioning model for PPL (Fig 2), with one school variable (PA policies) and one home-neighborhood variable (Recreational facilities) acting as potential mediators. The first subgroup comprised students in schools with PA policies ≤4 (*n* = 126), yielding a slope of *b* = 0.24, representing the weakest association between PA and PPL in contexts with limited institutional support. A second subgroup included those in schools with PA policies >4 and Recreational facilities ≤2.75 (*n* = 196), where the slope changed to *b* = 0.28. The participants in schools where PA policies >4 and Recreational facilities >2.75 (*n* = 74) displayed the most pronounced slope (*b* = 0.40). This suggests that the synergy between supportive school policies and accessible neighborhood facilities creates a more stimulating environment, significantly strengthening how PA translates into higher PPL. Essentially, when the physical environment provides more opportunities for practice, the positive impact of PA on PPL is maximized.

As shown in Table 2, when comparing the multiple linear models estimated for the entire sample with those obtained through recursive partitioning, the latter demonstrated a slightly better fit. For both MC and PPL, the model-based trees obtained higher Pearson correlation coefficients (with an improvement of *r* = 0.06–0.09) and lower estimation errors (i.e., MSE and MAE) than the multiple linear models. These performance metrics validate our initial hypothesis: the relationship between PA and the outcomes is not linear across all contexts, but is significantly enhanced by specific environmental thresholds. This also showcase that the recursive partitioning approach might provide a more nuanced and accurate understanding of how school and neighborhood characteristics influence children’s motor development.

**Table 2:** Performance parameters for model-based trees and linear models for motor competence and perceived physical literacy.

		Model-Based Tree	Linear Model
Motor Competence	Pearson R-value	0.44	0.35
	MSE	10.77	11.72
	MAE	2.62	2.79
Perceived Physical Literacy	Pearson R-value	0.55	0.49
	MSE	0.17	0.18
	MAE	0.32	0.33

MSE = mean squared error, MAE = mean absolute error.

## Discussion

Our findings support the study’s central hypothesis: while PA is positively associated with both MC and PPL, these relationships are not uniform across the sample but are significantly moderated by environmental contexts. Specifically, the model-based tree analysis revealed that school facilities, aesthetics, and PA policies—alongside neighborhood recreational facilities—identify distinct subgroups where the impact of PA is amplified or constrained. In particular, the discovery that optimal school environments can more than double the effect of PA on MC, and that the synergy between school and home-neighborhood factors maximizes PPL, confirms that the developmental benefits of movement are conditioned by specific environmental thresholds.

Children motor development is inherently shaped by a complex interplay of individual, social, and environmental factors, so that its research calls for a multidimensional and ecologically-grounded approach [20]. Within this framework, schools and residential neighborhoods emerge as critical environments in which opportunities for movement and perceptions of competence are either nurtured or constrained [27–30]. This study examined the moderating role of the physical environment—both school and residential neighborhood—on the relationships between PA engagement with MC and PPL in school-aged children, using a model-based tree approach. Despite the ever-increasing recognition of the role of the environment in shaping PA behaviors, few studies have examined how specific school and home neighborhood characteristics influence the strength of PA’s relationship with MC and PPL [46], while even less is known about how these contextual factors interact with one another or with sociodemographic variables such as gender and SES. Addressing this gap, our study contributes novel insights into how place-based factors can condition developmental trajectories (e.g., MC, PPL) in childhood.

The findings of the current study demonstrate that specific physical environmental features significantly moderate the strength of these relationships and that boys outperform girls in MC, PPL, particularly in the physical domain, and PA engagement. These findings are consistent with previous research [2,21], in which boys showed significantly higher levels of MC, PA, and PPL than girls. These differences may reflect gendered patterns of participation in PA and socialized beliefs around MC and self-perception [47]. Boys may be afforded more opportunities for active play and structured sports, which in turn can reinforce higher self-perceptions of competence [3,22]. These gender disparities highlight the need for gender-responsive interventions that promote equitable access and engagement in diverse forms of PA from an early age. To effectively reduce these gaps, interventions should move beyond traditional competitive sports models, which often favor male participation, and instead incorporate a broader range of non-competitive, cooperative, and creative physical activities. Furthermore, school-based programs could benefit from pedagogical strategies that challenge gender stereotypes and provide girls with targeted mastery experiences to bolster their self-perception of competence [48]. From an environmental perspective, designing school playgrounds with diverse ‘activity zones’—rather than predominantly sports-centric layouts—could encourage girls to engage more actively in movement without the pressure of male-dominated spaces.

The model-based trees provided novel insights into how specific environmental factors moderate the association between PA and both MC and PPL. For MC, school-based variables—namely sports and play facilities, school aesthetics, and PA policies—emerged as key moderators. Notably, children attending schools with higher-quality sports facilities and aesthetically appealing environments, coupled with comprehensive PA policies, exhibited stronger associations between PA and MC. This suggests that favorable school environments not only facilitate more PA but may also amplify the developmental benefits of such activity on motor skill acquisition [27,30]. The steepest slope observed (b = 2.07) for children in schools with high scores in all three areas underscores the importance of rethinking the synergistic physical environmental constructions such as the nexus of recreational infrastructure, aesthetics and policies in promoting PA engagement and MC.

In the case of PPL, both school and home-neighborhood environments served as moderators. PA policies again emerged as a significant school-level factor, while recreational facilities in the neighborhood context influenced the strength of the PA–PPL relationship. The positive moderation effect of these variables aligns with the existing literature on the ecological model of health behavior [23,37], in which access to diverse recreational spaces and supportive institutional policies may not only increase children’s opportunities for engagement in PA but also foster greater confidence and self-perception regarding their physical capabilities [9]. Interestingly, the combination of strong school policies and rich recreational access yielded the highest slope (b = 0.40). This emphasizes the interplay between institutional and community-level supports. Importantly, this study assesses PPL—children’s self-evaluation—rather than the actual PL as a holistic construct. While MC captures objective performance, PPL reflects the internalization of PL related-elements of movement and its importance. This distinction is vital, as subjective perceptions often drive long-term engagement independently of actual motor skill levels.

Our findings suggest that PPL, as a self-reflective construct, is deeply influenced by the variety and quality of the contexts where movement occurs. Unlike MC, which represents objective skill, PPL is built through the internalization of successful experiences in diverse settings. High-quality neighborhood facilities strengthen the PA-PPL link by providing non-evaluative spaces for autonomous exploration. In these environments, movement can be experienced as a personal achievement rather than a graded task, directly boosting physical self-confidence. The synergy between school policies and neighborhood resources further facilitates the transition from ‘doing’ to ‘believing’; while schools might provide institutional validation, the neighborhood might offer the ‘practice ground’ to internalize these capabilities. Consequently, in resource-limited environments, this relationship is weakened (as shown by our lower slopes) because children lack the diverse success scenarios needed to translate physical effort into a robust sense of personal capability.

When the moderator effect was examined according to the PL domain, particular nuances were obtained. For instance, the closeness to parks fostered the association between PA and PPL in the social domain (see S3 Fig), which in turn highlights the importance of providing opportunities for children to interact with their peers while autonomously actively playing to empower their PA-based social skills [49]. Additionally, school-based facilities such as drinking fountains and wildlife or nature gardens when coupled with aesthetically enhanced school environments were found to strengthen the link between PA and PPL both in the psychological and cognitive domains (see S2 and S4 Figs). These findings emphasize the need to reconsider how both school and neighborhood infrastructure policies are designed, as they collectively influence children’s holistic motor development and PL acquisition [50].

It is important to note that none of the environmental (school and neighborhood) moderators negatively impacted the association between PA with MC and PPL (which would depict a downhill path), rather it was these environment factors that slightly or strongly moderated the aforementioned association. These results are consistent with the hypothesis of this study, as in this region every school is obliged to fulfill its duty in promoting students’ health (Decree 106/2022). Consequently, school-based PA policies in the form of extracurricular PA, movement-based pedagogies, active transport, etc. are applied, which in turn not only contribute to fostering the students’ PA but also to their MC and PPL. This study thus showcases the importance of schools developing and integrating PA policies so that students can benefit by enhancing not only their level of PA engagement but also their actual competence and self-perception of PL, which can be considered a holistic perspective of health [50].

This study has several strengths in that it used a model-based recursive partitioning method, which allowed us to detect heterogeneity in how PA relates to developmental outcomes across different environmental contexts—an approach that goes beyond the limitations of traditional linear regression. Whereas traditional linear modeling often assumes homogeneity across populations, potentially masking meaningful subgroup-specific variations, model-based trees recursive partitioning enables the identification of the distinct environmental and contextual moderators that influence how PA relates to MC and PPL across different settings [44]. This method can reveal differential patterns that would otherwise be obscured in aggregate analyses [27,29]. The use of this analytical strategy is particularly relevant in PA, in which interactions between behavior and environment are rarely uniform. In doing so, it offers a more nuanced and ecologically valid understanding of how environmental factors shape the developmental outcomes related to movement and health in children [37]. The study also controlled for key covariates such as gender and SES, increasing the validity of the observed moderating effects.

However, certain limitations must be acknowledged. The findings should be interpreted with caution, as the study’s cross-sectional design precludes causal inference. Also, although self-reporting measures for PA and PPL are widely used and validated [8,39], they may be subject to recall bias or socially desirable effects. Furthermore, the study’s dropout rate must be considered, as attrition may have influenced the final sample’s representativeness. Regarding the variables, the limited variability observed in certain school factors (e.g., school grounds suitability) might have restricted the model’s ability to detect additional moderation effects. It is also important to acknowledge that using a purely quantitative approach may not fully capture the holistic and multidimensional nature of PL. Future research could benefit from mixed-methods designs to provide a more comprehensive understanding of children’s movement experiences. Moreover, the psychosocial and cultural factors that were not included in the models (e.g., peer norms, parental modeling) could also interact with environmental moderators. Future studies should adopt longitudinal designs and consider integrating psychological and social dimensions to better understand how complex systems interact over time.

From the point of view of practical implications, these findings offer actionable insights for school administrators, urban planners and policy makers. Enhancing the quality and accessibility of school-based physical environments—such as playgrounds, gymnasiums, and outdoor aesthetics—along with implementing supportive PA policies, can help optimize the impact of PA on children’s motor development and PPL. Likewise, investments in neighborhood recreational infrastructure, including parks, can complement school efforts and foster a more active, confident, and physically literate young generation. Importantly, these improvements should be equitably distributed to ensure safe and inclusive access for girls, specifically designing environments that challenge gender stereotypes and avoid exacerbating existing disparities between the socio-demographic groups.

## Conclusions

This study underscores the significance of physical environmental factors in moderating the relationship between PA and MC, as well as PPL in children. While the cross-sectional design precludes causal claims, our findings suggest plausible mechanisms through which the environment may amplify the benefits of PA. Using a model-based recursive partitioning approach [44], we identified how school and neighborhood features—such as PA policies, recreational access, and environmental aesthetics—moderate these relationships. This method allowed us to detect subgroup-specific patterns according to specific environmental variables that influence these relationships. The findings provide valuable insights for stakeholders to develop targeted inclusive interventions and policies that enhance opportunities for skill development and perceived competence regarding MC and PPL. In line with the ecological model of health behavior [23], our data indicate that supportive environments are associated not only with higher PA engagement but also with strengthened perceptions of children’s confidence, competence, and social interaction through movement [9,37]. This suggests that the environment might act as a catalyst; for instance, the synergy between school policies and neighborhood recreational facilities might provide the necessary ‘practice grounds’ to internalize movement as a successful experience, thereby reinforcing PPL. Compared to standard linear regression, which assumes a uniform relationship across the entire population, the model-based tree approach is particularly suited for detecting moderation as it automatically identifies complex interactions and non-linear effects. By uncovering specific environmental thresholds, this method allows for the identification of distinct subgroups where the impact of PA is significantly different, a task that would require pre-specified interaction terms in traditional models. The model-based tree approach thus offers a robust framework for exploring complex interactions and detecting heterogeneity in educational and public health research.

## Supporting information

S1 Fig Linear regression-based tree for Perceived Physical Literacy (PPL) in the physical domain. The terminal nodes of the tree plot report the intercept and estimate of physical activity (PA), but the models are controlled by gender (G) and socio-economic status (SES) as reported in equations. Variables related to the school environment are shown with a blue background, while those related to the home neighborhood are shown with a yellow background.
(TIF)

S2 Fig Linear regression-based tree for Perceived Physical Literacy (PPL) in the psychological domain. The terminal nodes of the tree plot report the intercept and estimate of physical activity (PA), but the models are controlled by gender (G) and socio-economic status (SES) as reported in equations. Variables related to the school environment are shown with a blue background, while those related to the home neighborhood are shown with a yellow background.
(TIF)

S3 Fig Linear regression-based tree for Perceived Physical Literacy (PPL) in the social domain. The terminal nodes of the tree plot report the intercept and estimate of physical activity (PA), but the models are controlled by gender (G) and socio-economic status (SES) as reported in equations. Variables related to the school environment are shown with a blue background, while those related to the home neighborhood are shown with a yellow background.
(TIF)

S4 Fig Linear regression-based tree for Perceived Physical Literacy (PPL) in the cognitive domain. The terminal nodes of the tree plot report the intercept and estimate of physical activity (PA), but the models are controlled by gender (G) and socio-economic status (SES) as reported in equations. Variables related to the school environment are shown with a blue background, while those related to the home neighborhood are shown with a yellow background.
(TIF)
